# Computed tomography evaluation of extensive intravenous angioleiomyoma: a case report

**DOI:** 10.1186/s12880-020-0417-2

**Published:** 2020-02-07

**Authors:** Rui Sun, Hongwei Guan, Hui Li, Yixuan Bai, Fei Wang, Changzhong Li

**Affiliations:** 1 Department of Obstetrics and Gynecology, Shandong Provincial Hospital Affiliated to Shandong University, 324 Jingwu Road, Jinan, Shandong 250021 People’s Republic of China; 2 Nursing Department, Shandong Provincial Hospital Affiliated to Shandong University, 324 Jingwu Road, Jinan, Shandong 250021 People’s Republic of China; 3 Department of Obstetrics and Gynecology, Shandong Provincial Hospital Affiliated to Shandong University, 324 Jingwu Road, Jinan, 250021 People’s Republic of China

**Keywords:** Three-dimensional CT reconstruction, Volume rendering reconstructions, Uterine angioleiomyoma, Intravenous leiomyomatosis, Surgery

## Abstract

**Background:**

Uterine angioleiomyoma is a rare variant of leiomyoma, and the main therapy is complete surgery. This study introduces the benefit of three-dimensional computed tomography reconstruction for preoperative preparation.

**Case presentation:**

A 50-year-old woman presented because of chest distress after activity, with worsening symptoms. After examination, the final diagnosis was uterine angioleiomyoma. The tumour originated in the uterus; grew into the right iliac vein; coursed along the iliac vein, inferior vena cava, and right atrium; and finally invaded the right ventricle. To best complete the surgery, a multidisciplinary surgery was selected. Before the surgery, a three-dimensional computed tomography reconstruction model was created to assess the tumour status, and this model enabled the surgery to be completed successfully.

**Conclusion:**

Three-dimensional computed tomography reconstruction is of great significance for the preoperative diagnosis of uterine angioleiomyoma and the formulation of surgical treatment plans. Based on its vivid images, surgeons can perform operations more effectively and safely.

## Background

Uterine angioleiomyoma, also called intravenous leiomyomatosis (IVL), is a rare variant of leiomyoma and is a benign tumour. It usually occurs in middle-aged women after myomectomy, originates from either a uterine leiomyoma or the venous wall of uterine veins, and grows within the venous channels or lymphatic vessels without invading them [1]. However, we have found that the morbidity of the disease is increasing and that the increasing rate of IVL diagnoses is due to improvements in medical technology, diagnostic techniques, and knowledge of this disease [2].

There are no specific manifestations of this disease. Patients may have menorrhagia or an abdominal mass, and the symptoms depend on where the tumour grows. Patients usually fail to feel the tumour until it reaches the heart and causes severe clinical symptoms. IVL is difficult to diagnose with clinical and radiological proof; only after histopathologic examination can a definite diagnosis be made [3]. The tumour varies in size and position and always has a complicated shape, growing from the primary site to the blood vessel and then following the blood flow. Angioleiomyoma has been proven to be oestrogen-dependent [4], and consequently, the treatment depends on the patient’s age and whether she has the desire to conceive [5].

To obtain more pre-operative anatomic information and make the surgical procedure safer and smoother, we considered three-dimensional computed tomography (CT) reconstruction using a CT volume rendering (VR) technique to be the best approach. Recently, we successfully removed an angioleiomyoma with the help of CT three-dimensional reconstruction.

## Case presentation

A 50-year-old woman went to Shandong Provincial Hospital affiliated to Shandong University because of chest distress after activity. The patient had experienced this distress for 6 months without obvious precipitating factors or accompanying symptoms, and the symptoms disappeared at rest. Before she came to our hospital, the symptoms of chest distress had increased significantly for 1 month; the farthest distance the patient could walk was up to 50 m, and walking was accompanied by a heavy feeling in the lower limbs and an uncomfortable feeling of abdominal distension. Cardiac ultrasound revealed a successional mass in the right atrium and right ventricle, which originated from the inferior vena cava and extended to the pulmonary artery. The mass in the right atrium was approximately 3*5 cm in size, and the mass in the right ventricle was approximately 7 cm in length. A pelvic ultrasound revealed a high echo mass at the posterior wall of the uterus and uterine leiomyomas. The patient had had hypertension for 1 year, denied the presence of other diseases, and had a normal menstrual history. She did not have more children because of the family planning policy but did have a son and a daughter.

**Fig. 1:**
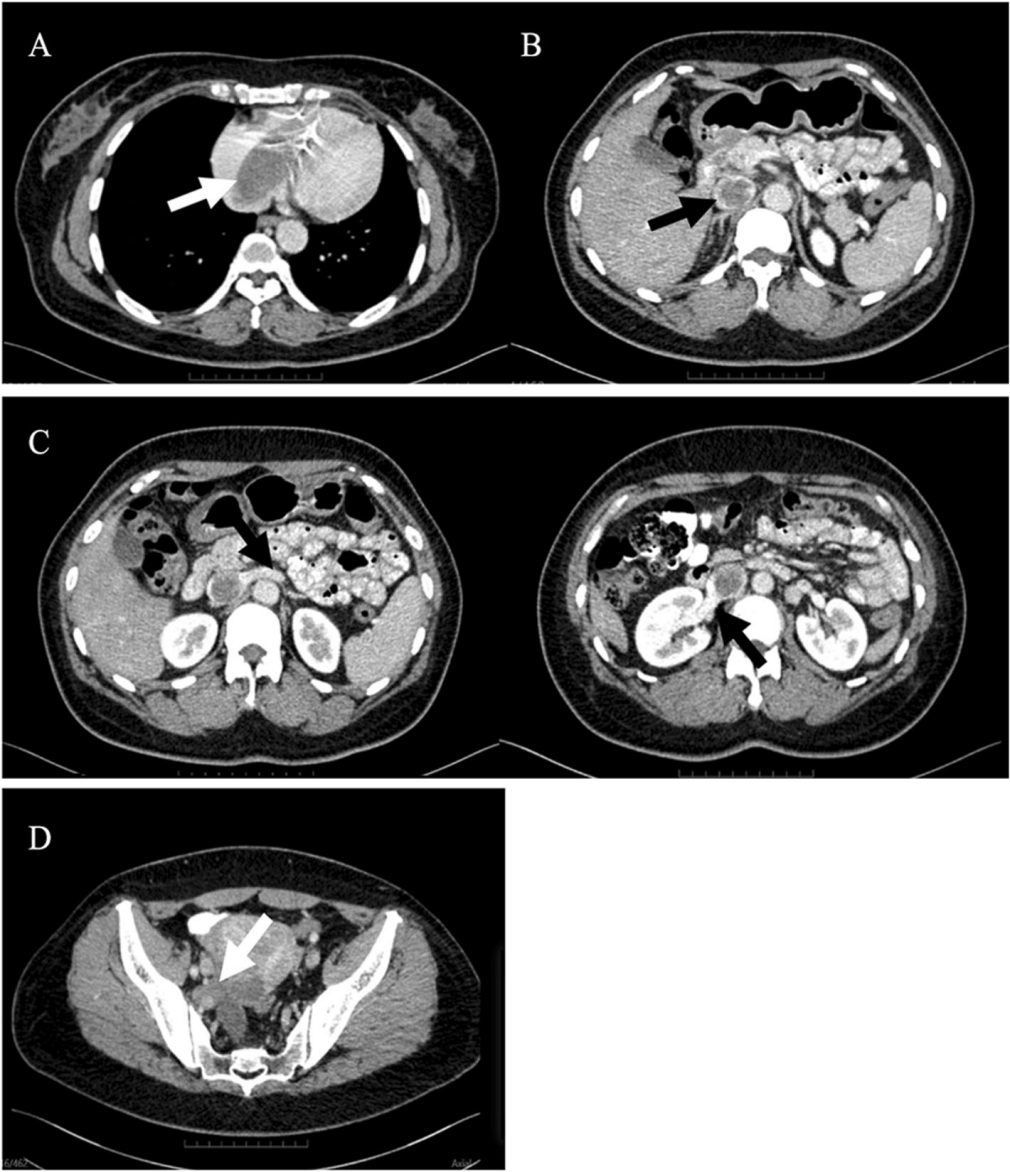
Axial slice of the CT image shows the shape and route of extension of the intravenous leiomyomatosis. **a** This image shows that the tumour grew into the right atrium and extended to the right ventricle; **b** This image shows that the tumour grew along the inferior vena cava, resulting in enlargement of the vein; **c** These two images show that the bilateral renal veins were not invaded; **d** This image shows that the leiomyoma grew into the vein through the right uterine vein and internal iliac vein

**Fig. 2:**
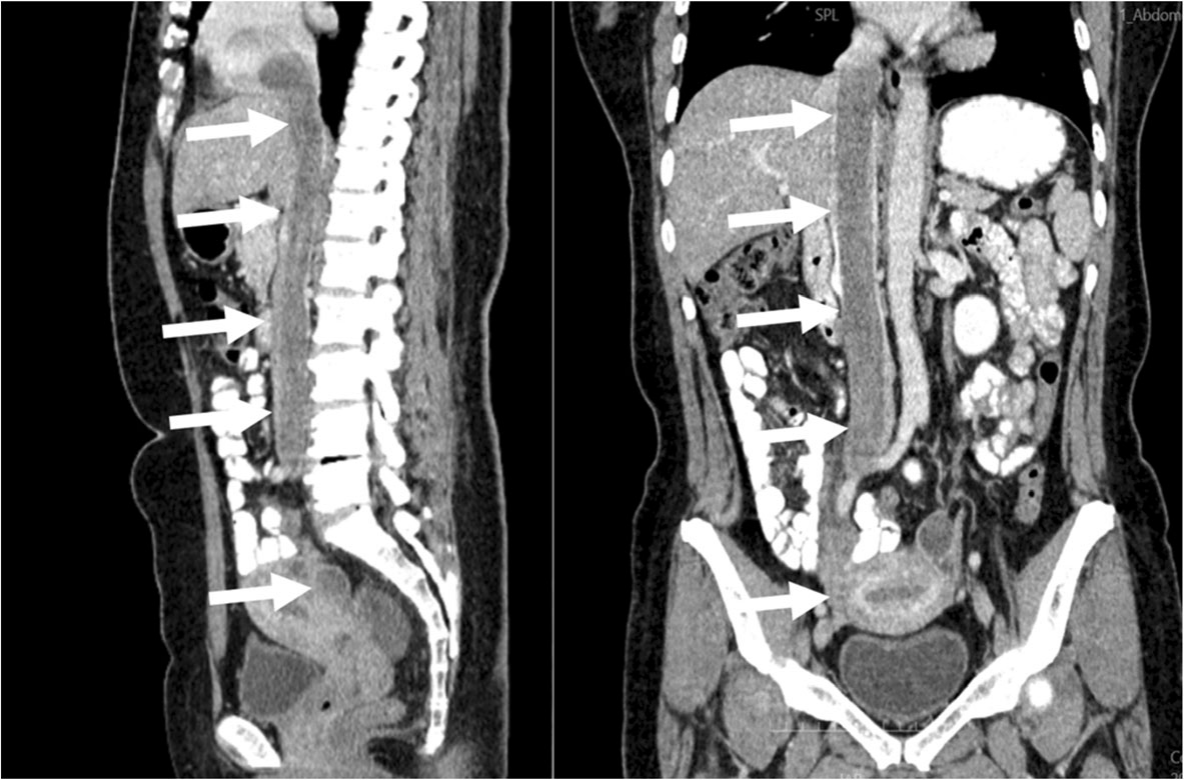
Sagittal and coronal slices of the CT multiplanar reformation show almost the whole appearance and travel path of the tumour; there was a continuous mass coursing through the inferior vena cava to the heart

**Fig. 3:**
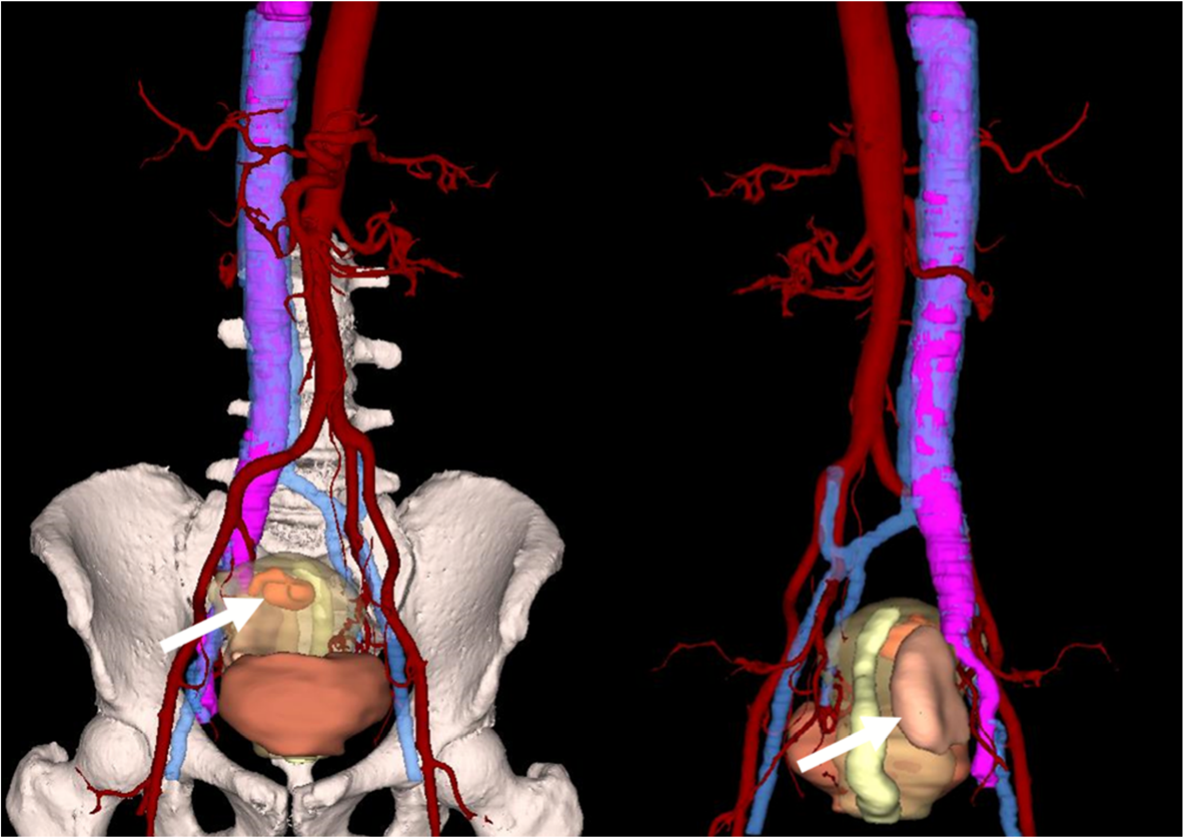
A three-dimensional reconstruction image using the VR technique clearly shows the overall appearance of the tumour, the extent of invasion, the location of the tumour invading the blood vessel, the starting and ending location, the travel path, and the tumour dissemination. (The arrows point to the tumour inside (left) and behind (right) the uterus, and the purple tissue is the tumour growing inside the venous system)

Next, to make an accurate diagnosis, it was suggested that she undergo a CT examination. The CT acquisitions were performed on a SOMATOM Definition AS CT scanner (SIEMENS) with the following settings: 120-kVp tube voltage, 300-mA tube current, 1.5-mm slice thickness, and 512 × 512 imaging matrix. The enhanced scan was performed by the mass injection method, and the contrast agent Iopamidol Injection (universum 370 mg I/ml) was injected through the cubital vein with a double-cylinder high-pressure syringe at a dosage of 1.5 ml/kg and an injection rate of 3.0 ml/s. The patient underwent plain scanning plus double-phase enhanced scanning (28 s in the arterial stage and 65 s in the venous stage). An abdominal contrast-enhanced CT revealed a 3.7 * 4.3 cm lesion in the uterus and a right iliac vein - iliac vein - inferior vena cava (Fig. 1b) - right atrium (Fig. 1a) - right ventricular filling defect. The bilateral renal veins were not invaded (Fig. 1c), and the tumour may have grown into the blood vessels through the right iliac vein (Fig. 1d). Gynaecological-related tumour markers were within the normal range. After all of these examinations, the patient was considered to have a uterine myoma with right iliac vessel metastasis and right iliac vein to right atrial tumour thrombus formation. After a multidisciplinary discussion, the patient was eventually diagnosed with IVL and required multidisciplinary surgery. Before the procedure, to facilitate the surgical resection and improve the accuracy of the operation, CT multiplanar reformation (MPR) (Fig. 2) and CT VR reconstruction (Fig. 3) were used to evaluate the tumour. With the help of extracorporeal circulation technology, a one-stage surgery was finally recommended.

**Fig. 4:**
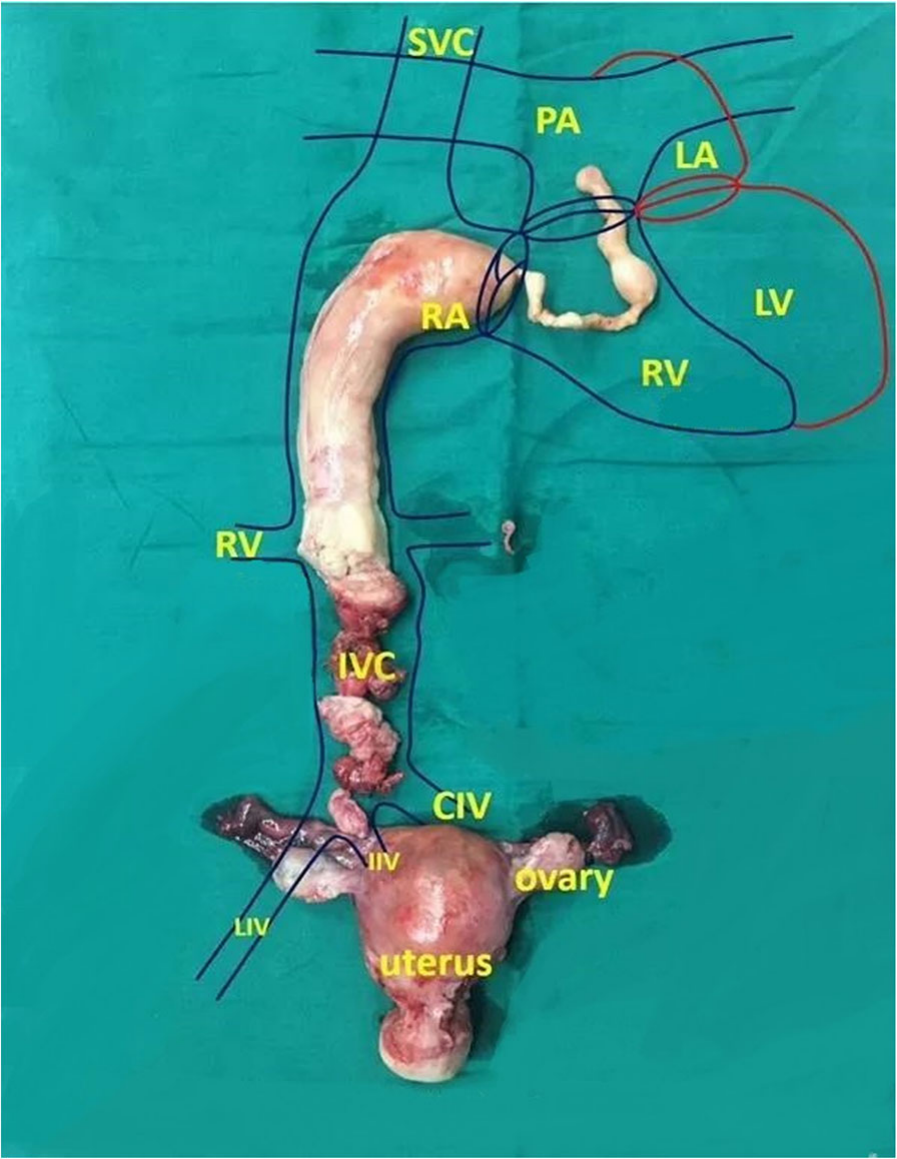
The final shape of the tumour and the relationship between the tumour and the circulatory system

After successful anaesthesia, the patient was placed in a supine position. The sternum was opened longitudinally, followed by the pericardium, free ascending aorta, and superior and inferior vena cava, and a set of blocking belts was applied as a backup. The right uterine vein was dilated after the laparotomy and initial adhesiolysis and was observed to be the canal where the leiomyoma grew into the right iliac vein. A total abdominal hysterectomy and bilateral salpingo-oophorectomy were performed. The right internal iliac vein was retrogradely separated from the uterine artery, and the internal iliac vein was resected from the bifurcation. The common iliac vein and inferior vena cava were separated to the lower edge of the liver, and a set of blocking belts was applied. After extracorporeal circulation was established, a small 3-cm opening was made from the right atrium to block the inferior vena cava blood flow, and a 7-cm incision was made from the inferior vena cava. By pulling the tumour down, it was completely delivered from the inferior vena cava. The heart cavity and venous lumen were examined, and there were no tumour remnants. At this point, the tumour was completely removed (Fig. 4).

**Fig. 5:**
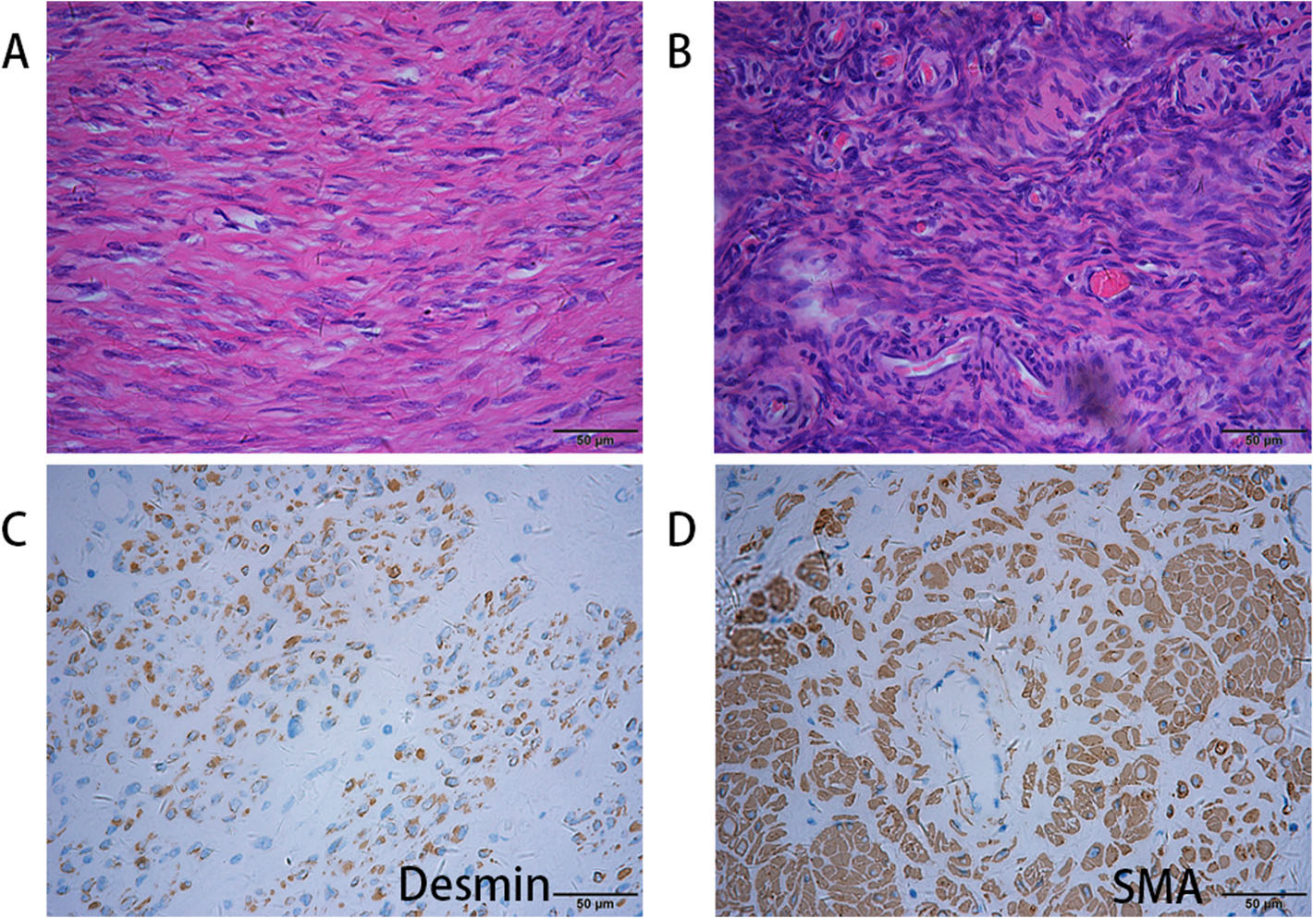
Microscopic picture of the patient. The tumour consisted of spindle cells (**a**), which encircled the blood vessels as they grew (**b**). Immunohistochemically, the tumour was positive for Desmin (**c**) and SMA (**d**), confirming its myogenic origin. It was negative for p53. The Ki-67 level was approximately 1%, indicating low cell division activity. The final diagnosis was IVL

The postoperative pathology indicated multiple uterine leiomyomas and inferior vena cava leiomyomas, and the immunostaining showed desmin +, smooth muscle actin (SMA) +, P53 -, and Ki-67 + (1%). Combined with the immunostaining results, more than two experienced pathologists diagnosed the patient as having IVL (Fig. 5).

## Discussion and conclusion

Uterine angioleiomyoma is a benign tumour of the mesoderm. It was first reported by Brich-Hirschfeld in 1896 [6], and they found that it can spread from the uterus into blood vessels and grow along the direction of blood flow. In 1907, Durck reported the first case of intracardiac extension and named it intracardiac leiomyomatosis (ICL) [7]. In addition to the involvement of veins, lymphatic vessels can also be affected, and thus, the condition is called intravenous leiomyomatosis (IVL).

IVL is common in women of childbearing age. Early studies found that approximately 0.25–0.4% of patients with uterine fibroids were diagnosed with IVL [7, 8]. Early clinical manifestations of IVL lack specificity. IVL may cause compression symptoms, such as changes in the menstrual cycle or menstrual volume, lower abdominal or back pain, and so on [3]. As the tumour grows, it can cause orthostatic hypotension and syncope due to obstruction of blood reflux. When it invades a pelvic blood vessel, a neoplasm or even a free mass is formed; a series of obstructive symptoms may occur, and patients can even develop a life-threatening embolism. IVL grows along the blood vessels and travels through the uterine vein or the ovarian vein, the iliac vein, and the inferior vena cava, ultimately arriving at the right atrium of the heart or even the right pulmonary vessel [9]. The aetiology of IVL has not been clarified to date, and its pathogenesis may involve factors such as oestrogen, progesterone, growth factors, cytokines, extracellular matrix components, etc.

The key to treating the disease is complete surgical removal of the tumour, with or without hysterectomy and bilateral adnexectomy, depending on the patient’s age and fertility requirement, via a one- or two-stage procedure in case of thoracic involvement. The use of extracorporeal circulation made one-stage surgery the best choice because of the low pulmonary embolism risk, reduced pain, protection of the vital organs and avoidance of haemodynamic complications. However, two-stage surgery, involving resection of the abdominal/pelvic and intrathoracic components in two operations within a two- to six-week interval time, may lessen coagulopathy and haemorrhage caused by the long operation time and extent of injury. In our case, a one-stage operation meant a shorter surgical and anaesthesia time, a briefer hospital stay, and a faster recovery period, which were more beneficial. Although the amount of blood loss in the first stage of the operation is high, there is no significant difference in the rate of allogeneic blood transfusion and postoperative complications between one- and two-stage surgery groups [10].

Analysis of the pathological features showed that although IVL is a histologically benign type of tumour, it has an unusual growth pattern similar to that of malignant tumours. Based on the relationship between muscles and vascular cavities, IVL is classified into 3 histologic types: capillary or solid, cavernous, and venous [11]. Under a microscope, we can see that it is composed of smooth muscle cells with varying degrees of hyperplasia and that the spindle muscle cells encircle the blood vessels as they grow [3]. Merchant found peculiar vessels within the vessel architecture, which might be important in the pathogenesis of IVL [12]. The blood supply may affect the degeneration of the far end of the tumour [4]. The recurrence rate of IVL is 16.6–30% [13]. Patients with total hysterectomy, bilateral salpingo-oophorectomy and all visible tumours excised had lower recurrence rates (7.6%) than patients with a simple myomectomy (33.3%) [14]. Therefore, complete surgery is the key to treating IVL. Strict follow-up should also be performed for patients with incomplete tumour resection and ovarian preservation. Since IVL is a hormone-dependent disease [15], the use of tamoxifen, medroxyprogesterone, gonadotropin-releasing hormone agonists (GnRH-a) and so on [8, 16–20] may be useful in preventing its recurrence.

Imaging examination is helpful in the diagnosis and treatment of IVL. Ultrasound (US), CT, fluorine-18-fluorodeoxyglucose positron emission tomography/computed tomography (^18^F-FDG PET/CT), magnetic resonance imaging (MRI), echocardiography (ECG), and intraoperative trans-oesophageal ultrasound (TEU) possess different advantages. Ultrasound is a convenient and fast way to measure the tumour size and nature. In the ultrasound image, IVL appears as a hypoechoic mass near the cervix or uterus with a clear boundary and many large tubular or fissure-free echogenic areas inside with no blood flow detected. When the tumour invades the vein, it is discovered as a beaded, medium-echo mass. Although ultrasound has high clinical diagnostic value, the diagnostic compliance rate of multi-slice CT is superior to that of ultrasound. On CT imaging, IVL is an irregularly shaped mass with uneven density and uneven nodular enhancement. When it extends to the heart, the appearance of the head and the large tail is similar to a “walking stick head” or “snake head”. Preoperative CT diagnosis of an angioleiomyoma is extremely difficult, since previous case reports did not provide common CT characteristics. However, the role of CT imaging remains essential in the differential diagnosis of this condition [13, 21]. CT imaging can effectively facilitate the clinical diagnosis and evaluation of the curative effect of treatment, and in particular, three-dimensional reconstruction before surgery guides the way. The benign nature of IVL makes it difficult to diagnose by ^18^F-FDG PET/CT. PET/CT shows low uptake of ^18^F-FDG and offers glycometabolic information on the lesion, but it can also facilitate the identification of unexpected foci of the tumour [22]. On MRI imaging, the IVL signals are similar or slightly higher than those of muscle on T1WI and higher than those of muscle on T2WI. There is great contrast between soft tissues and the tumour on MRI, which makes the relationship between the tumour and vascular tissue clear. MRI has the advantages of non-ionizing radiation and multi-sequence, multi-angle and multi-parameter imaging, and it has great importance in diagnosing, guiding surgery and performing prognostic evaluations [23]. However, an MRI examination has a long acquisition time, a slow imaging speed, a thick layer, and poor reconstruction, which limits its application. TEU guided the complete removal of an intracardiac leiomyoma in a surgical procedure without vein injury. Intraoperative TEU plays a significant role in helping to plan the surgical approach, monitor the movement of the tumour and the inferior vena cava during the extraction, and assess the completeness of the tumour resection [24].

In this case report, the guiding significance of CT three-dimensional reconstruction in the surgical treatment of this disease was discussed. Multi-spiral CT can scan a wide range of positions in a short time. Apart from the time advantage, CT multiplanar reformation can also provide valuable information. The image data from contrast-enhanced CT scanning were saved in the Digital Imaging and Communications in Medicine (DICOM) format using special software to obtain the reconstruction image. After three-dimensional reconstruction, the image can clearly show the overall appearance of the tumour, the extent of invasion, and the relationship between the lesion and the neighbouring organs. Although the accuracy rate of VR is slightly reduced, the location and spread of lesions may be more stereoscopic. VR can show the location of the tumour invading the blood vessel, the starting and ending location, the travel path, the dissemination, and other features, providing more detailed, vivid and stereoscopic results for the clinic. The imaging data have laid a good foundation for the successful implementation of the surgical plan, which has an important clinical application value.

For gynaecologists in primary hospitals in some developing countries, the diagnosis of uterine lesions, such as the most common uterine fibroids, adenomyosis, and endometrial cancer, often relies on ultrasound rather than CT images. Therefore, these physicians are often not proficient or accurate at reading conventional CT images. For relatively rare diseases such as uterine vascular leiomyoma, their interpretation of the size and location of the lesions is even more inaccurate. In any case, the VR images show less detail and are less reliable than the raw CT images, but is nevertheless useful for providing an generally overview of the disease extent. That has been well-established for 3D images of the skull, for example, whereby the reconstruction tends to be more reliable, but is not to be interpreted by itself. VR can provide gynaecologists with an intuitive three-dimensional image before surgery, which would greatly help them formulate accurate surgical plans and establish surgical confidence. Surgeons can make specific surgical plans for the patient based on these images, including the approaches and procedures of the surgery and the best way to remove the tumour thoroughly, save time and reduce tentative procedures. Overall, the final benefits are clear, as CT three-dimensional reconstruction imaging can make the excision more accurate, reduce surgical trauma and prevent surgical complications.

IVL is a rare variant of leiomyoma. Three-dimensional CT reconstruction is of great significance for the diagnosis and treatment of IVL. Compared with other imagological examinations, three-dimensional CT reconstruction can provide more detailed, vivid and stereoscopic images. Based on such vivid images, surgeons can perform operations more effectively and safely.

## Data Availability

The datasets generated and/or analysed during the current study are not publicly available due to the patients’ privacy but are available from the corresponding author upon reasonable request.

## Abbreviations

^18^F-FDG PET/CT: Fluorine-18-fluorodeoxyglucose positron emission tomography/computed tomography
CT: Computed tomography
DICOM: Digital Imaging and Communications in Medicine
ECG: Echocardiography
GnRH-a: Gonadotropin-releasing hormone agonists
ICL: Intracardiac leiomyomatosis
IVL: Intravenous leiomyomatosis
MPR: Multiplanar reformation
MRI: Magnetic resonance imaging
TEU: Trans-oesophageal ultrasound
US: Ultrasound
VR: Volume rendering
